# Implantable collamer lens versus small incision lenticule extraction for high myopia correction: A systematic review and meta-analysis

**DOI:** 10.1186/s12886-021-02206-9

**Published:** 2021-12-27

**Authors:** Kai Cao, Jingshang Zhang, Jinda Wang, Mayinuer Yusufu, Shanshan Jin, Shuying Chen, Ningli Wang, Zi-Bing Jin, Xiu Hua Wan

**Affiliations:** Beijing Institute of Ophthalmology, Beijing Tongren Hospital, Beijing Key Laboratory of Ophthalmology and Visual Sciences, Capital Medical University, No17, Hougou ally, Dongcheng district, Beijing, 100005 China

**Keywords:** Intraocular lens, Implantable collamer lens, small incision lenticule extraction, high myopia

## Abstract

**Purpose:**

To compare the efficacy, safety, predictability and visual quality between implantable collamer lens (ICL) implantation and small incision lenticule extraction (SMILE) for high myopia correction in adults.

**Methods:**

A systematic review and meta-analysis was conducted. A comprehensive literature search was done based on databases including PubMed, Science Direct, Embase, and the Cochrane Central Register of Controlled Trials. The efficacy index, safety index, changes in Snellen lines of corrected distance visual acuity (CDVA), predictability (difference between post-operative and attempted spherical equivalent error, SER), incidence of halos, and change in higher-order aberrations (HOAs) were compared. Mean difference (MD) and 95% confidence interval (CI) was used to estimate continuous outcomes, risk ratio (RR) and 95%CI was used to estimate categorical outcomes.

**Results:**

Five observational studies involving 555 eyes were included in this review. Studies’ sample sizes (eyes) ranged from 76 to 197. Subjects’ refraction ranged from -6 diopter (D) to -12D. Study duration of most researches were 6 months or 12 months. Compared to SMILE, ICL implantation showed better efficacy index (MD=0.09, 95%CI:0.01 to 0.16) and better safety index (MD=0.08, 95%CI: 0.00 to 0.16). Compared with SMILE, more ICL-treated eyes gained one or more Snellen lines of CDVA (RR=1.54, 95%CI:1.28 to 1.86), more gained two or more lines (RR=2.09, 95%CI:1.40 to 3.13), less lost one or more lines (RR=0.17, 95%CI:0.05 to 0.63). There was no difference in predictability between two treatments, RRs of predictability of within ±0.5D and ±1D were 1.13 (95%CI: 0.94 to 1.36) and 1.00 (95%CI: 0.98 to 1.02). Compared with SMILE, ICL implantation came with a higher risk of halos [RR=1.79, 95%CI: 1.48 to 2.16] and less increase in total HOAs (MD=-0.23, 95%CI: -0.42 to -0.03).

**Conclusion:**

Compared with SMILE, ICL implantation showed a higher risk of halos, but equal performance on SER control, and better performance on efficacy index, safety index, CDVA improvement and HOAs control. Overall, ICL implantation might be a better choice for high myopia correction in adults.

## Introduction

Nowadays, it is well accepted to use small incision lenticule extraction (SMILE) to correct low-to-moderate myopia [1, 2]. For high myopia correction, SMILE also brought satisfying prognosis [3, 4], however, SMILE has its inherent limitations such as thick ablation depths, limited ablation zones and increased aberrations, SMILE could increase the risk of dry eye [5], myopic regression [6], haze [7] and corneal ectasia [8].

Implantable collamer lens (ICL) implantation was another promising way for high myopia correction [9], the postoperative spherical equivalent error (SER) was reported to be predictable [10, 11] and stable [12, 13]. ICL implantation broadened the scope of target population, such as patients with thin cornea [14] and even keratoconus [15]. A few studies compared ICL implantation with SMILE for high myopia correction, conclusions from different studies were controversial. Siedlecki J [16] and coworkers reported ICL implantation yielded better uncorrected distance visual acuity, better refractive accuracy, and fewer higher-order aberrations (HOAs) than SMILE. Moshirfar M [17] and coworkers thought SMILE might be comparable to ICL for high myopia correction, similarly, Wei R [18] reported both treatments showed compared performance for high myopia correction.

In this review, we aim to make a strengthened comparison between ICL implantation and SMILE for high myopia correction in adults. The efficacy index, safety index, changes in Snellen lines of corrected distance visual acuity (CDVA), predictability, incidence of halos, and change in higher-order aberrations (HOAs) would be compared between two treatments.

## Methods and Materials

### Inclusion criteria

Studies were included under the following consideration: 1) Subjects with high myopia (SER should be equal or greater than -6 diopter, D); 2) Subjects’ age≥18 years old; 3) The intervention measures must include ICL implantation and SMILE.

### Exclusion criteria

Studies were excluded in any of the following condition: 1) Studies of case report, letter, comment or review; 2) Studies used only ICL implantation or only SMILE; 3) Studies that included low to moderate myopic patients (SER of -0.5D to -6D) or other kinds of patients.

### Databases and Search strategy

We searched PubMed, Science Direct, and the Cochrane Central Register of Controlled Trials (from inception to 15 August, 2021) for studies published in English, the detailed search strategies were shown in the end of the manuscript.

### Outcomes

According to the scale of extracted data, the following outcomes were quantitatively assessed: The efficacy index, safety index, changes in Snellen lines of CDVA, predictability, incidence of halos, and change in HOAs. Besides, the following outcomes were qualitatively described: endothelial cell loss, complications including cataract and dry eye, visual quality including objective scatter index (OSI) and modulation transfer function cut-off frequency (MTF_cut-off_) value.

### Data Extraction

The following information measured at last-follow up time of each study was extracted: First author, publication year, subjects’ mean/median age, sample size (number of eyes), study design, mean follow-up duration, subjects’ SER range and type of ICL. We extracted the data of outcomes for analysis using a pre-designed data form. Briefly, for categorical data, such as the number of halos, the number of events were extracted. For continuous data, such as the efficacy index, we extracted the mean value and standard deviation (SD).

### Data synthesis and statistical analysis

Meta-analysis was performed using either a fixed-effects model or a random-effects model according to the heterogeneity across included studies. The heterogeneity was assessed by a Q-test and the I^2^ statistic. The I^2^ statistic describes the percentage of variability caused by heterogeneity rather than by chance. An I^2^ of ≤50% indicates a relatively small heterogeneity across studies, subsequently a fixed-effects model would be used, otherwise a random-effects model would be used [19]. We used the mean differences (MDs) and 95% confidence intervals (CIs) to make comparison of continuous outcomes between ICL implantation and SMILE, we used risk ratios (RRs) and 95% CIs to estimate categorical outcomes. We used Egger’s test to determine publication bias. The significance level was set to be 0.05, two-tailed. All statistical analysis was done using the open-source R program (Version 4.0.0).

## Results

### Paper selection

**Fig. 1:**
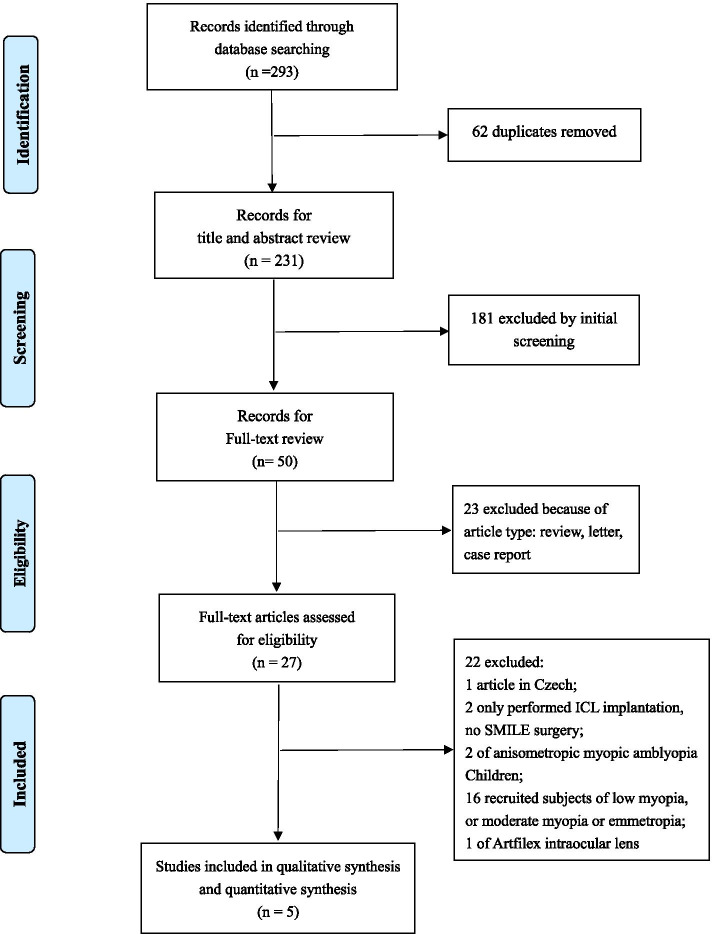
Flow chart of paper selection

The paper selection process was shown in Figure 1. Initially, a total of 293 articles were identified from PubMed, Science direct and Cochrane Central Register of Controlled Trials. 62 duplicates were removed, then 181 publications were further excluded by title and abstract. 23 reviews, letters or case reports were excluded. 22 articles were further excluded due to the following reasons: study in Czech (n=1), studies where only ICL implantation was performed (n=2), studies where subjects were children (n=2), studies recruited subjects of low or moderate myopia, or emmetropia (n=16), studies of iris-fixated Artiflex lens (n=1). Finally, 5 studies [13, 16, 18, 20, 21](involving 555 eyes) were included in this meta-analysis.

### Characteristics of the included studies

The characteristics of the five included studies were shown in Table 1. All studies were observational studies, and were published between year 2019 to 2021. All subjects were high myopic adults (age≥18), the sample sizes (number of eyes) ranged from 76 to 197. Subjects’ refraction ranged from -6D to -12D. Three studies used EVO Visian ICL (Visian ICL V4c) and two studies used Visian ICL without knowing the model. The median follow-up duration of five studies ranged from three months to 60 months.

**Table 1:** Characteristics of included studies

First author	Publish Year	Age (years)		N (eyes)	Study design	Follow-up time (months)	Myopia range		ICL type
		ICL	SMILE				ICL	SMILE	
Qin Q	2019	20 to 34	20 to 31	96	Observational study	Median: 3	-6.25D to -10D	-6.25D to -10D	Visian ICL(V4c)
Niu LL	2020	27.3±5.5	28.4±4.2	76	Observational study	Median: 12	-6D to -9D	-6D to -9D	Visian ICL(V4c)
Siedlecki J	2020	33.9±6.4	32.2±7.6	80	Observational study	ICL: 27.8 ± 14.3 SMILE: 26.6 ± 17.7	-6D to -10D	-6D to -10D	Visian ICL
Wei R	2020	27.0±5.3	28.7±5.0	197	Observational study	Median: 6	-6D to -10D	-6D to -10D	Visian ICL(V4c)
Jiang Z	2021	26.8±5.2	28.3±5.3	106	Retrospective case series	Median: 12	-6D to -12D	-6D to -12D	Visian ICL

ICL: implantable collamer lens. SMILE: small incision lenticule extraction

### Efficacy and safety index

**Fig. 2:**
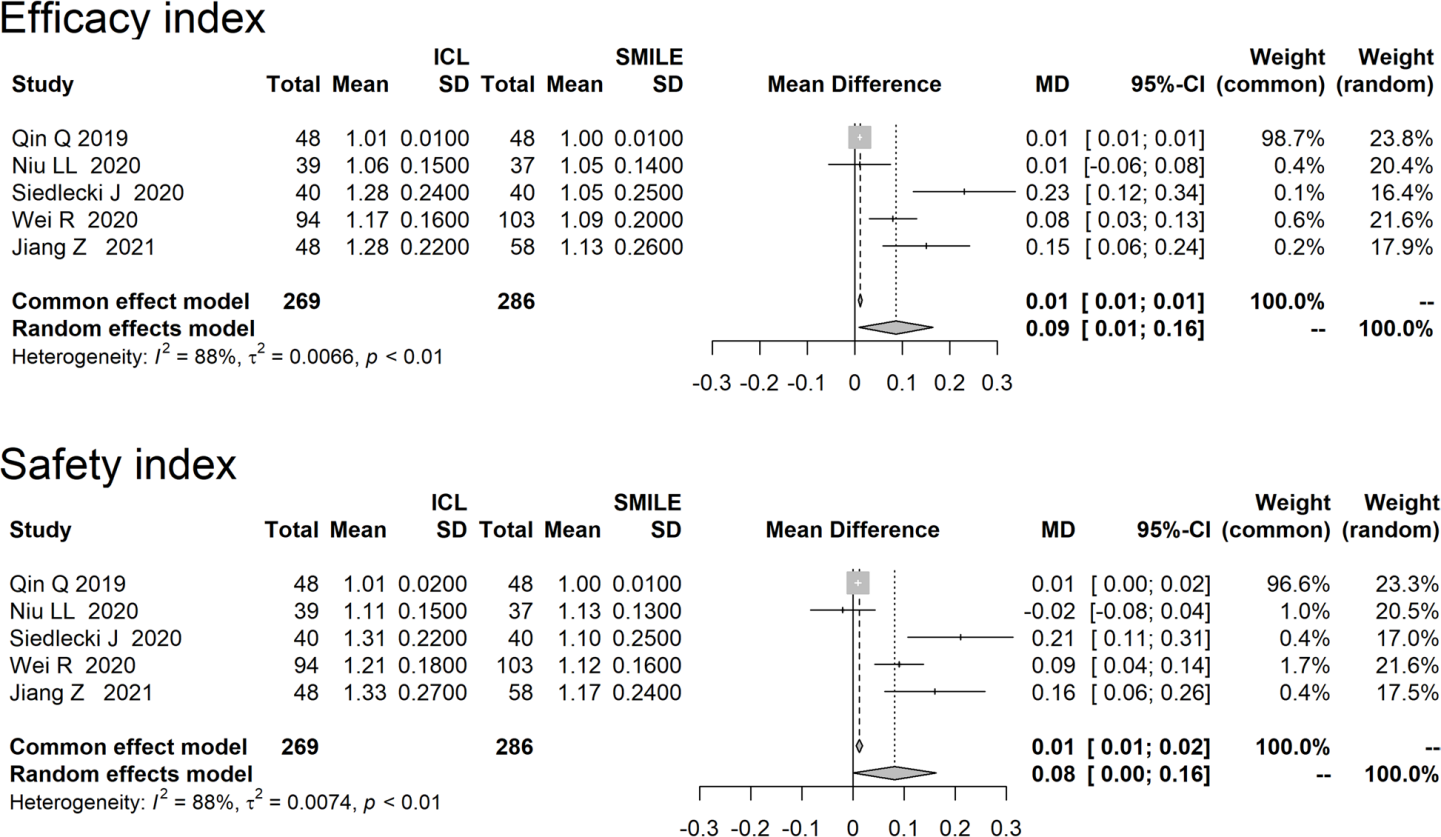
Forest plot of comparison on efficacy index and safety index

All five studies evaluated the efficacy index (Figure 2). Overall, ICL implantation showed a statistically better efficacy index (MD=0.09, 95%CI:0.01 to 0.16) and a better safety index (MD=0.08, 95%CI: 0.00, 0.16) than SMILE. Random-effects model were used for meta-analysis due to a large heterogeneity across studies (I^2^>50%).

### CDVA

**Fig. 3:**
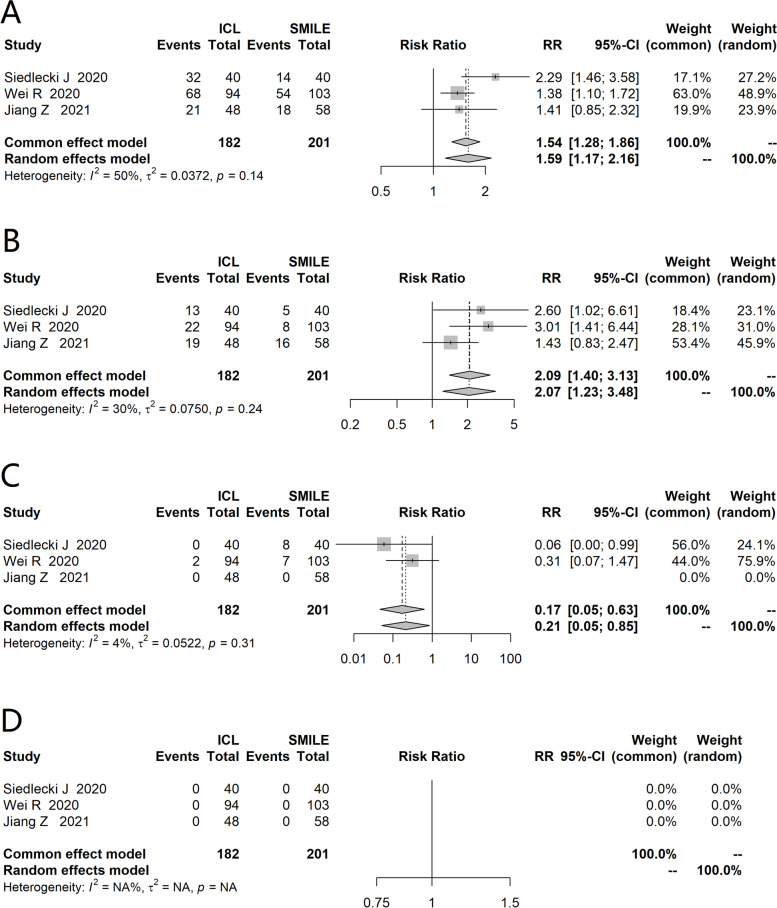
Forest plot of comparison on gaining or loss of corrected distance visual acuity (A: gaining one or more lines, B: gaining two or more lines, C: losing one or more lines, D: losing two or more lines)

Three studies [16, 18, 20] assessed change in Snellen lines of CDVA (Fig. 3), the heterogeneity across three studies was small (I^2^=50%), so a fixed-effects model was applied. Compared with SMILE-treated eyes, more ICL-treated eyes gained one or more lines of CDVA (RR=1.54, 95%CI:1.28 to 1.86), more ICL-treated eyes gained two or more lines of CDVA (RR=2.09, 95%CI:1.40 to 3.13), less ICL-treated eyes lost one or more lines of CDVA (RR=0.17, 95%CI: 0.05 to 0.63). No eyes lost two or more Snellen lines in each group.

### Predictability

**Fig. 4:**
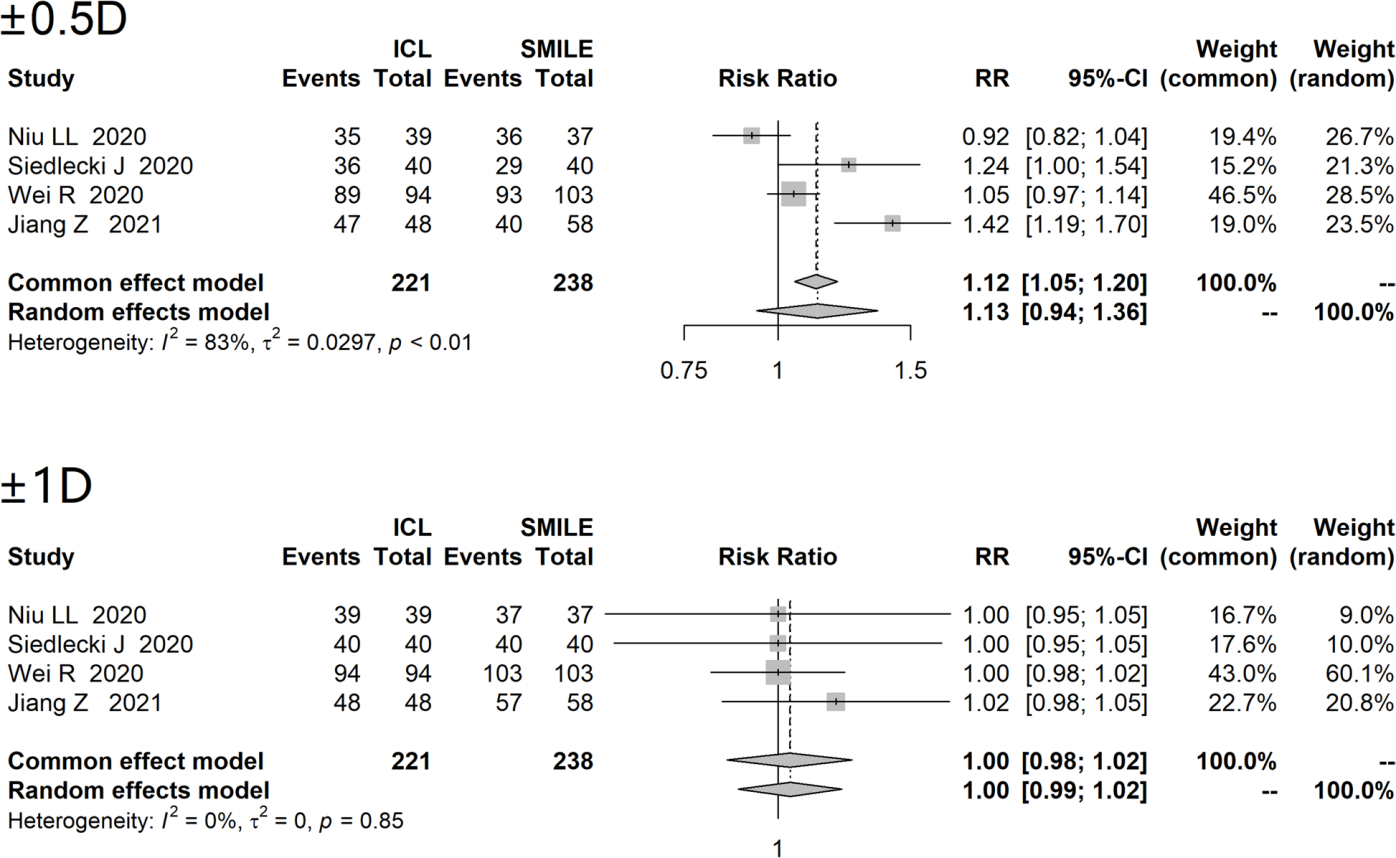
Forest plot of comparison on predictability

Four studies (except for study of Qin Q [21]) reported predictability of both treatments, difference of post-operative SER and attempted SER was used to evaluate the predictability. The I^2^ of predictability of within ± 0.5D and within ± 1D were 80% and 0% respectively, thus a random-effects model and a fixed-effects model were used to conduct meta-analysis respectively. Forest plot (Fig. 4) showed no statistical difference in predictability of within ±0.5D between two treatments (RR=1.13, 95%CI: 0.94 to 1.36) , there was no statistical difference in predictability of within ±1D either (RR=1.00, 95%CI: 0.98 to 1.02).

### Halos

**Fig. 5:**
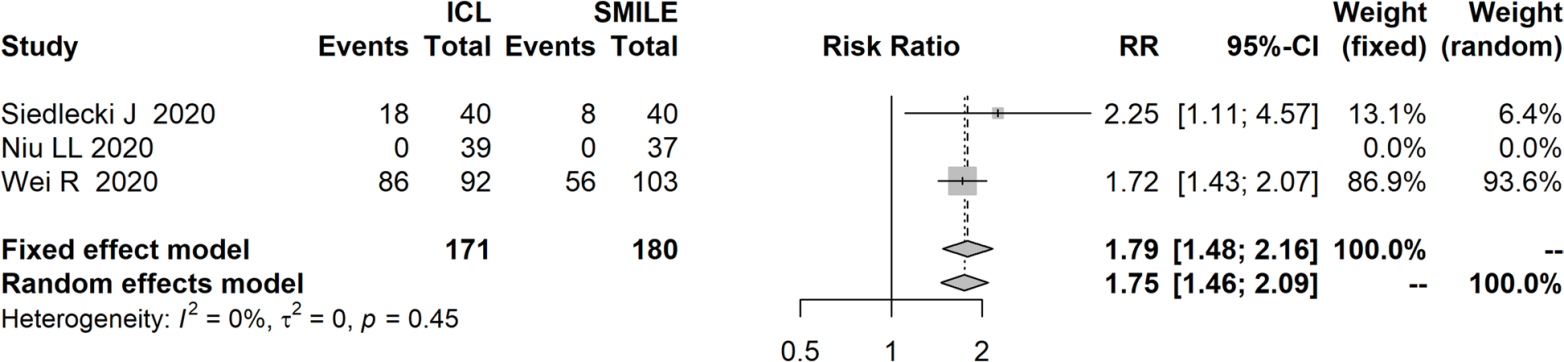
Forest plot of comparison on halos

Three studies [13, 16, 18] evaluated halos after treatment, ICL implantation showed statistically higher risk of halos than SMILE (Fig. 5), the RR was 1.79 (95%CI: 1.48 to 2.16), there was no heterogeneity across included studies (I^2^=0%).

### Increase of total HOAs

**Fig. 6:**
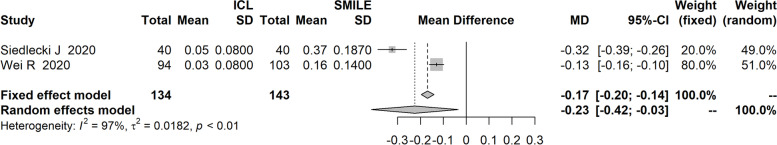
Forest plot of comparison on total higher-order aberrations

Two studies assessed change of total HOAs, the heterogeneity across two studies was large (I^2^=97%), thus a random-effects model was applied. ICL-treated eyes showed a smaller increase of total HOAs (Fig. 6) than SMILE-treated eyes (MD=-0.23, 95%CI: -0.42, -0.03).

### Other outcomes

For other outcomes including cornea endothelial cell loss, complications including cataract and dry eye, visual quality including objective scatter index (OSI) and modulation transfer function cut-off frequency (MTF_cut-off_) value, no pooled MD or pooled RR was calculated due to limited data. Qin Q and coworkers reported no significant endothelial cell density loss in either ICL-treated eyes or SMILE-treated eyes. Niu LL [13] and coworkers reported no statistical difference between ICL implantation and SMILE in change of either OSI or MTF_cut-off_ before and after surgery. None study reported occurrence of cataract or dry eye in each treatment group.

### Publication bias test

Publication bias was checked using Egger’s test, no publication bias was found by Egger’s test (p>0.05).

## Discussion

High myopia, predisposed by genetic and environmental factors [22–24], poses challenge to clinical treatment, clinicians have to balance the benefit and risk since no choice is perfect. In this meta-analysis, we compared two popular options (SMILE and ICL implantation) for high myopia correction to help doctors make better decision. Efficacy index, calculated from visual acuity, is the most important outcome reflecting the efficacy of treatments. By this review, ICL implantation showed a better efficacy index than SMILE, which indicated that ICL implantation might be more efficient than SMILE for high myopia correction. ICL implantation also showed a better performance on safety index than SMILE. Besides, ICL implantation tended to come with a lower risk of visual acuity loss than SMILE, only 1.09% (2/182) of ICL-treated eyes lost one or more lines of CDVA, while the percentage reached 7.46% (15/201) in SMILE-treated eyes.

The predictability on SER reflects the accuracy of treatments, both ICL implantation and SMILE should achieve good accuracy according to their design principles. In this meta-analysis, both treatments showed good and equal performance on predictability, 93.67% (207/221) ICL-treated eyes and 83.19% (198/238) SMILE-treated eyes achieved a predictability of within ±0.5D, 100% (221/221) and 99.58% (237/238) achieved a predictability of within ±1D. Many previous studies reported similar findings, usually more than 95% ICL-treated eyes were within ±1.00 D of the intended refraction [11, 25–27]. A large retrospective study included 722 SMILE-treated high myopic eyes, 98% were within ±1.00 D of the intended refraction [28].

This meta-analysis showed ICL implantation leads to a smaller change in total HOAs than SMILE, this is reasonable since theoretically ICL implantation does not cause damage to the physiological structure of the cornea itself, thus eyeballs are able to preserve the adjustment ability, ensuring that subjects could obtain a more ideal visual quality. On the contrary, SMILE causes damage to the corneal surface morphology, and thus leads to change of HOAs. Evidence from previous studies supported the theory, this meta-analysis showed a smaller increase in total HOAs in ICL-treated eyes compared with SMILE-treated eyes, many other studies reported similar findings, usually, there was no significant increase of HOAs ICL-treated eyes [29, 30], while for SMILE-treated high myopic eyes, significant increase of total HOAs were commonly reported [31–34].

Contrast sensitivity, OSI, and MTF_cut-off,_ are also important reflections of visual quality, however, no quantitative conclusion was drawn in this meta-analysis because few studies assessed these parameters. None of the included study performed contrast sensitivity test, Igarashi A [35] and coworkers reported that ICL implantation improved the contrast sensitivity while SMILE decreased the contrast sensitivity, Shin JY [36] and coworkers reported that ICL implantation induced fewer ocular and corneal HOAs, which resulted in a better contrast sensitivity at mesopic levels. Current evidence [36] showed no difference on OSI between ICL implantation and SMILE. But in terms of MTF_cut-off_ value, evidence was contradicted, Qin Q [21] and coworkers reported that the postoperative MTF_cut-off_ value of ICL-treated eyes was higher than SMILE-treated eyes, while Niu LL [13] and coworker found no significant difference between two treatments. However, given that either study of Qin Q [21] or study of Niu LL [13] was observational studies, which meaning the preoperative MTF_cut-off_ value of two treatments might not be balanced, thus assessing the change in MTF_cut-off_ value and change in OSI might be more meaningful, Niu LL [13] and coworkers reported no significant change before and after surgery, either for MTF_cut-off_ value or OSI.

Complications are also important reflections of treatments’ safety, endothelial cell density loss was thought to be a main backward of ICL implantation [37]. In this meta-analysis, Qin Q’s study [21] reported no significant decrease before and after surgery either in the ICL group or SMILE group, the current evidence is not enough to assess the safety of both treatments. Halos were reported to be the leading complication of ICL implantation, the prevalence rates ranged from 15.2% [38, 39] to 93.5% [16, 18]. By this meta-analysis, up to 60.81% (104/171) ICL-treated eyes and 35.56% (64/180) SMILE-treated eyes perceived halos, in study of Wei R [18], the halos rates in ICL group and SMILE group were even as high as 93.5% and 54.4%, the reason might that the follow-up time was short (six months), as halos were commonly seen in the early period after ICL and SMILE surgery. Although no cataract formation was reported in either ICL-treated eyes or SMILE-treated eyes in five studies, it doesn’t represent that cataract is not a concern since the follow-up duration was usually one year or less except for study of Siedlecki J [16] (about two-year follow-up), Guber I^39^ and coworkers reported the lens opacity rate could reach 40.9% in 133 high myopic eyes 10 years after ICL implantation, 18 eyes of which underwent phacoemulsification.

In conclusion, both ICL implantation and SMILE had satisfying and equal performance on refraction control. ICL implantation came with a higher risk of halos, but was better than SMILE on efficacy, safety, CDVA improvement and total HOAs control. Overall, for high myopia correction, ICL implantation might be a better choice than SMILE. However, the conclusion came from observational studies with relatively short-term follow-up, evidence from randomized controlled trials and long-term studies is still needed.

## Search strategy

1. **PubMed, Science direct, Embase**

((posterior chamber phakic intraocular lens) OR piol OR (implantable collamer lens) OR ICL OR ticl OR V4 OR V4C OR STAAR) AND ((small-incision lenticule extraction) OR smile OR (cornea refractive surgery)) AND (myopia OR (refractive errors) OR refraction)

2. **Cochrane Central Register of Controlled Trials**

#1posterior chamber phakic intraocular lens

#2piol

#3implantable collamer lens

#4ICL

#5ticl

#6V4

#7V4C

#8STAAR

#9#1 or #2 or #3 or #4 or #5 or #6 or #7 or #8

#10small-incision lenticule extraction

#11smile

#12 cornea refractive surgery

#13#10 or #11 or #12

#14myopia

#15refractive errors

#16refraction

#17 #14 or #15 or #16

#31#9 AND #13 AND #17

## Data Availability

Data would be available upon reasonable request
